# Phosphate Tailings and Clay-Based Ceramic Membranes: Tailoring Microstructure and Filtration Properties via Alkali Activation

**DOI:** 10.3390/membranes15020052

**Published:** 2025-02-05

**Authors:** Amine El Azizi, Hanane El Harouachi, Dounia Ahoudi, Soundouss Maliki, Mohammed Mansori, Mohamed Loutou

**Affiliations:** 1 Laboratory of Molecular Chemistry, Materials and Environment (LCM2E), Multidisciplinary Faculty of Nador (FPN), Mohammed First University, B.P. 300, Selouane 62700, Morocco; elazizi.amine@ump.ac.ma (A.E.A.); hanane.elharouachi@ump.ac.ma (H.E.H.); dounia.ahoudi@ump.ac.ma (D.A.); soundouss.maliki@ump.ac.ma (S.M.); 2 IMED-Lab, Laboratory of Innovative Materials, Energy and Sustainable Development, Faculty of Science and Technology, Cadi Ayyad University (UCA), Marrakech 40000, Morocco; m.mansori@uca.ac.ma

**Keywords:** ceramic membranes, phosphate tailings, clay lithologies, alkali-activated materials, porosity tailoring, wastewater filtration

## Abstract

The increasing demand for sustainable water treatment technologies has driven the development of advanced ceramic membranes with tailored properties. This study explores the fabrication of ceramic membranes using phosphate tailings and clay lithologies as alternative raw materials, offering a sustainable and cost-effective approach to membrane production. The focus is on tailoring membrane porosity through the deposition of multilayered alkali-activated coatings, leveraging geopolymerization chemistry to enhance structural and functional performance. The manufactured ceramic membranes were investigated using X-ray fluorescence spectrometry, X-ray diffraction, thermogravimetric analysis, Fourier transform infrared spectroscopy, scanning electron microscopy, and a filtration test pilot. Results revealed the suitability of both phosphate tailing and the clay for membrane processing, while alkali activation effectively modulates the membrane’s porosity (from 1–10 μm to 0.1–1 μm) and mechanical strength (up to 20 MPa). Both tailored and untailored membranes demonstrated favorable performance. Key findings include the formation of a well-interconnected pore network and improved compressive strength, which resulted in sustained filtration performance under challenging operational conditions. The membranes demonstrated their suitability for environmental and industrial applications by achieving high efficiency in industrial effluent filtration tests.

## 1. Introduction

The increasing global demand for clean water, driven by rapid urbanization, industrial growth, and population expansion, has placed immense pressure on freshwater resources. According to recent reports, nearly two billion people worldwide face water scarcity, and this number is expected to grow due to the impacts of climate change and unsustainable water management practices [1,2,3,4]. Hydric stress is particularly acute in arid and semiarid regions, where natural water resources are insufficient to meet societal needs [5]. In parallel, the proliferation of industrial activities has led to the discharge of untreated wastewater, laden with harmful pollutants, into aquatic systems [6]. This pollution exacerbates the scarcity of potable water and poses significant risks to ecosystems and public health. Addressing these challenges requires innovative solutions that prioritize water reuse, efficient wastewater treatment, and the adoption of sustainable technologies [7,8,9].

Among the emerging technologies, ceramic membranes have shown significant promise in advanced water filtration and purification systems. These membranes are valued for their robustness, thermal stability, and superior filtration performance compared to polymeric alternatives [10,11,12,13,14]. However, conventional ceramic membrane production relies heavily on virgin raw materials, such as kaolin and zircon [15], which are not only costly but also contribute to environmental degradation through mining and processing activities [16]. To align ceramic membrane production with sustainable development goals, the use of alternative raw materials, particularly industrial and mining waste, has garnered significant attention [17,18,19].

In recent years, researchers have explored a wide range of alternative raw materials to fabricate ceramic membranes. Industrial byproducts, including fly ash, blast furnace slag, and red mud, have also been used as precursors in membrane production, leveraging their aluminosilicate-rich composition [20,21]. Additionally, natural clays, volcanic ash, and diatomite have been incorporated into ceramic formulations to enhance membrane performance while reducing reliance on nonrenewable resources [22]. These studies demonstrate that waste-derived materials can effectively replace traditional feedstocks, offering both environmental and economic benefits. However, challenges remain in optimizing the microstructure, porosity, and mechanical properties of these membranes to meet the stringent requirements of industrial applications [23].

Phosphate tailings, a byproduct of phosphate ore processing, and clay lithologies represent underutilized resources with high potential for application in ceramic membrane manufacturing. Phosphate tailings are rich in silicates, alumina-bearing phases, and other mineralogical components suitable for ceramic production [24]. Meanwhile, clay lithologies provide natural aluminosilicates that are abundant, affordable, and widely available [25]. Leveraging these waste materials not only mitigates the environmental issues associated with their disposal but also provides an opportunity to create high-value products from industrial residues [26].

A critical aspect of ceramic membranes is their porosity, which determines their filtration efficiency, permeability, and fouling resistance. To enhance these properties, recent advancements have focused on tailoring membrane porosity through surface modifications. One such innovative approach involves the deposition of multilayered coatings of alkali-activated materials on ceramic substrates [27,28]. Alkali activation, a process rooted in geopolymerization chemistry, allows for precise control over the porosity and pore structure of the membranes [29]. This technique also improves the mechanical stability and chemical resistance of the membranes, broadening their applicability in water and wastewater treatment processes [30].

Compared to other sustainable membrane fabrication methods, such as phase inversion techniques for polymeric membranes using green solvents [31] or bio-based membrane production utilizing renewable polymers like chitosan and cellulose [32], the use of ceramic membranes derived from industrial waste offers distinct advantages. These include superior thermal and chemical resistance, longer operational lifespans, and the potential to operate under extreme conditions. Furthermore, methods such as electrospinning for polymeric membranes have shown promise in enhancing membrane functionality but often require high-energy inputs and specialized equipment [33]. In contrast, the utilization of waste-derived ceramic precursors, coupled with alkali activation, presents a cost-effective and environmentally friendly pathway. This approach not only minimizes the ecological footprint, but also aligns with circular economy principles by valorizing industrial byproducts. These attributes position waste-based ceramic membranes as a compelling alternative in the quest for sustainable water treatment solutions.

This study investigates the sustainable fabrication of ceramic membranes using phosphate tailings and clay lithologies as primary raw materials. It also explores the application of alkali-activated multilayered coatings to achieve tailored porosity and enhanced functional performance. The integration of these waste-derived materials into ceramic membrane manufacturing supports a circular economy approach, reducing reliance on virgin resources and contributing to the management of industrial byproducts. By addressing both environmental challenges and technological advancements, this research aims to provide a pathway for the development of cost-effective, high-performance ceramic membranes tailored to meet the demands of modern water treatment systems.

## 2. Materials and Methods

### 2.1. Materials

The primary raw materials used for the production of ceramic membranes included phosphate tailings (PT), chloritic clay (CC), and cork oak bark (CB), selected for their availability, composition, and functional properties.

Phosphate mine tailings were sourced from the phosphate mining region of Khouribga, a major production site for phosphate rocks in Morocco. As a byproduct of the beneficiation process, these tailings consist predominantly of silica and alumina-rich compounds, with minor amounts of calcium and iron oxides. The material was air-dried to remove surface moisture, followed by oven drying at 105 °C for 24 h. It was then pulverized in a ball mill to achieve a uniform particle size of less than 80 μm and sieved to ensure consistency. The chloritic clay was obtained from deposits located in nearby Safi City, Morocco, recognized for its abundance of aluminum silicate minerals, particularly chlorite. The raw clay was air-dried and subjected to thermal treatment at 105 °C to eliminate free moisture. It was then ground and sieved to achieve a particle size similar to that of the phosphate tailings. The mineralogical composition was analyzed using X-ray diffraction (XRD) to confirm its suitability for ceramic processing. Cork oak bark, serving as a pore-forming agent, was collected from trees grown in the Rif Mountains, Morocco. The bark, a renewable material rich in organic compounds, was oven-dried at 80 °C to remove residual water. It was subsequently ground into fine particles (<100 μm) to facilitate even dispersion within the ceramic mix. During the firing process, the organic matter in the bark would burn out, leaving behind interconnected pores.

### 2.2. Membrane Fabrication

The ceramic membranes were prepared by mixing the phosphate tailings and chloritic clay in equal proportions (50 wt% each) with cork oak bark added as a pore-forming agent at 10 wt% relative to the total mass of the clay and tailings. The dry components were homogenized in a planetary mixer to ensure a uniform blend. Deionized water, equivalent to 20–25 wt% of the total dry mass, was gradually added to the mixture to form a workable paste. The paste was shaped into flat disc membranes using a uniaxial pressing method at a pressure of 20 MPa. This ensured uniform density and thickness across all samples, critical for maintaining consistency during subsequent thermal treatments.

The shaped membranes were air-dried for 24 h at room temperature to eliminate surface moisture and prevent cracking. This was followed by oven drying at 105 °C for an additional 24 h to remove residual water. The dried membranes were then subjected to a two-step firing process in a programmable furnace. The first step involved heating to 600 °C at a controlled rate of 5 °C/min, holding at this temperature for 1 h to ensure complete burnout of the cork oak bark, which formed the desired pore structure. The second step involved sintering at the range 900–1000 °C for 2 h to consolidate the ceramic structure and achieve mechanical stability. The furnace cooling process was carried out gradually to prevent thermal shock and cracking.

To further enhance the surface properties and functionality of the fabricated membranes, a geopolymer coating was applied using a spray-coating technique. The geopolymer slurry was prepared by combining metakaolin with an alkaline activating solution comprising sodium hydroxide (NaOH) and sodium silicate (Na_2_SiO_3_) at a carefully controlled ratio to achieve optimal viscosity for spraying. The prepared slurry was loaded into a pneumatic spray gun equipped with a fine nozzle to ensure uniform application.

Before coating, the membranes were pretreated by cleaning their surfaces to remove any loose particles and drying at 105 °C. The spray-coating process was conducted in a controlled environment to ensure even distribution of the geopolymer slurry. Multiple thin layers were applied sequentially, allowing a brief air-drying period of 15–20 min between each layer to minimize the risk of cracking and to promote adhesion. The coating process was repeated until the desired layer thickness and surface coverage were achieved.

After the application, the coated membranes were cured at ambient temperature for 24 h to initiate the geopolymerization process, followed by thermal curing at 80 °C for 6 h to enhance the mechanical and chemical stability of the separation layer. This spray-coating approach provided a uniform and smooth geopolymer coating, effectively reducing surface defects and allowing for fine-tuning of the pore structure and surface chemistry, thereby improving the membranes’ performance in filtration applications.

### 2.3. Experimental Techniques

The fabricated membranes were thoroughly characterized to assess their mineralogical, microstructural, and functional properties. Characterization tests were conducted on both powdered raw materials and sintered membrane samples to evaluate their properties. X-ray fluorescence (XRF) analysis was performed using an Epsilon 4 spectrometer to determine the elemental composition of the materials. X-ray diffraction (XRD) was carried out with an Empyrean PANalytical diffractometer (Malvern, UK) equipped with copper Kα radiation (λ = 1.5418 Å). The diffractograms were recorded at room temperature over a 2θ range of 5°–70°, with a step size of 0.01°. Fourier transform infrared spectroscopy (FTIR) was performed using a Bruker Vertex 70 spectrophotometer (Billerica, MA, USA) in the range of 4000–400 cm^−1^. The samples for FTIR analysis were prepared as KBr pellets, consisting of 99 mg of potassium bromide mixed with 1 mg of the powdered sample. Thermogravimetric analysis (TGA) was conducted on a Setaram Setsys 24 instrument (Caluire-et-Cuire, France) under atmospheric conditions. The heating program involved a rate of 10 °C/min, using alumina as the reference material, with the temperature ramped up to a maximum of 1000 °C. The morphology and pore structure of the sintered membranes were examined using a Schottky Field Emission Scanning Electron Microscope (FE-SEM) (Nova NanoSEM 650, FEI Company, Hillsborough, OR, USA). This instrument was equipped with an energy-dispersive X-ray spectroscopy (EDS) system (TEAM™ integrated EDS with an Apollo X silicon drift detector, Pleasanton, CA, USA) for quantitative microanalysis. Prior to imaging, the samples were coated with a conductive layer to enhance resolution and minimize charging.

The membranes’ filtration performance was evaluated using distilled water and industrial effluent. The filtration experiments were conducted following a protocol adapted from previous studies [13,31], using a laboratory-scale filtration setup. Each membrane formulation was tested in triplicate to ensure reliability, with results reported as arithmetic means. Before filtration, the membranes were preconditioned by soaking in distilled water for 24 h to achieve consistent flow performance. The industrial effluent, provided by a metallurgical facility, was analyzed for key physical and chemical properties such as pH, turbidity, and chemical oxygen demand (COD) both before and after filtration. The changes in these parameters following filtration were monitored, quantified, and discussed in detail.

## 3. Results

### 3.1. Raw Material Characterization

**Figure 1:**
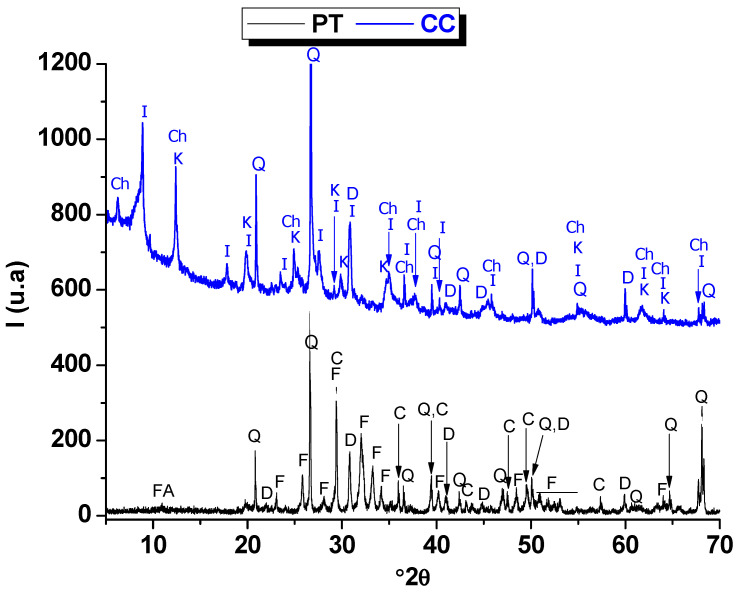
X-ray diffractograms of the phosphate tailings (PT) and chloritic clay (CC). F: fluorapatite; Q: quartz; D: dolomite; C: calcite; I: illite; K: kaolinite; Ch: chlorite.

Figure 1 shows the XRD diffractograms of the used raw materials. Phosphate tailings XRD pattern (Figure 1) suggests the presence of fluorapatite, dolomite, quartz, calcite, and dolomite as the primary mineral phases. These minerals are common in natural phosphate tailings and demonstrate both phosphatic and silicate mineral contributions. The chloritic clay XRD pattern (Figure 1) reveals chlorite as the most dominant mineral phase, with significant contributions from quartz, illite, kaolinite, and dolomite. Such mineralogy is in agreement with the chemical composition established in Table 1.

**Figure 2:**
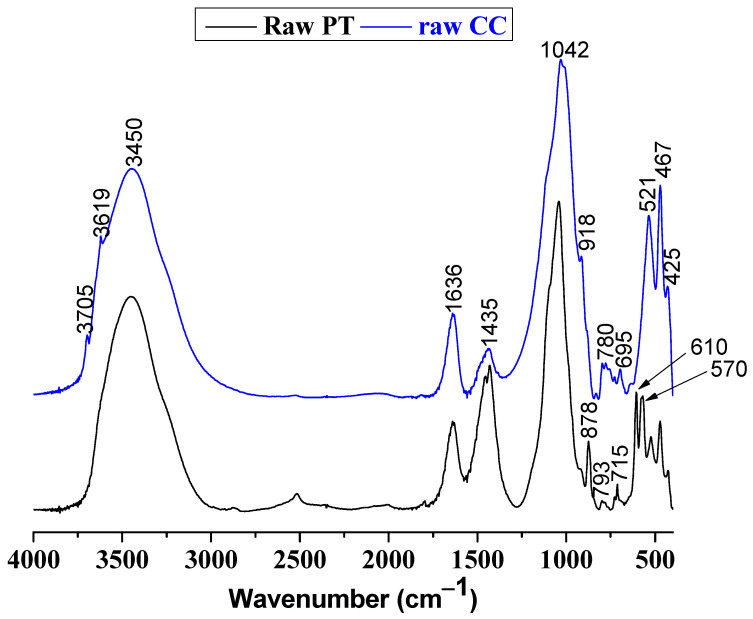
FTIR spectra of the employed raw materials.

For a more exhaustive comprehension, FTIR analysis was performed, and its results are displayed in Figure 2. In the phosphate tailings, the broad band at 3450 cm^−1^ is attributed to the O-H stretching of adsorbed water. A prominent band at 1636 cm^−1^ indicates H-O-H bending vibrations, confirming the presence of physically adsorbed water. The sharp band at 1435 cm^−1^ is linked to C-O stretching vibrations from carbonate minerals such as calcite or dolomite. The most significant peak at 1042 cm^−1^ corresponds to Si-O stretching vibrations, characteristic of quartz minerals. In the low-wavenumber region, bands between 610–425 cm^−1^ reflect Si-O and Al-O bending vibrations, confirming the presence of silicates and phosphate-related phases. For chloritic clay, the peaks at 3705 cm⁻¹ and 3619 cm^−1^ similarly corresponded to hydroxyl stretching vibrations, typical of clay minerals such as chlorite. The broad peak at 3450 cm^−1^ represents adsorbed water, while the band at 1636 cm^−1^ further confirms the presence of molecular water. The carbonate group’s C-O stretching vibration appears at 1435 cm^−1^, indicating minor carbonate impurities. A prominent band at 1042 cm^−1^ is associated with Si-O stretching vibrations, which are dominant in silicate minerals present in chloritic clay. In the lower wavenumber range, bands at 918 cm^−1^, 878 cm^−1^, 798 cm^−1^, 696 cm^−1^, 524 cm^−1^, 467 cm^−1^, and 425 cm^−1^ confirm Si-O and Al-O vibrations, typical of layered silicates like chlorite.

### 3.2. Thermal Behavior Microstructural Changes

**Figure 3:**
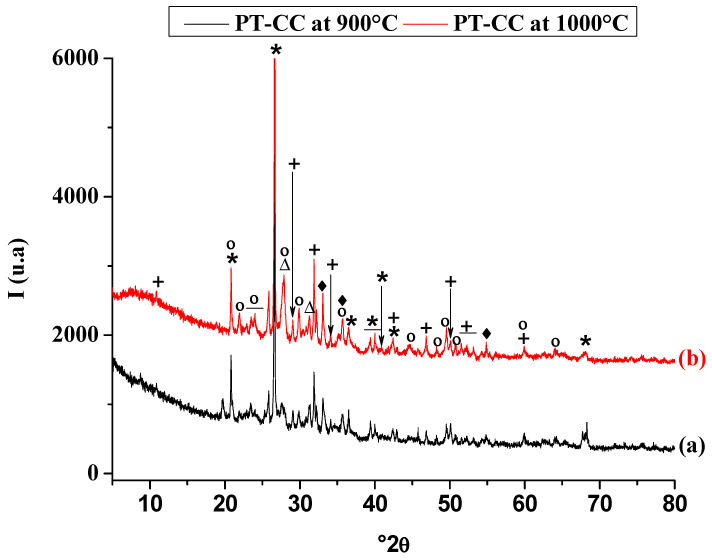
X-ray diffraction patterns of membranes of the studied blends heated at 900 °C (a) and 1000 °C (b). *: quartz; °: labradorite; +: fluorapatite; Δ: Orthoclase; ♦: Hematite; ◊: Labradorite.

Based on the X-ray diffraction patterns presented in Figure 3, significant thermal transformations occurred within the unfired PT-CC blends upon heating to 900 °C. The structural frameworks of chlorite and kaolinite, the predominant clay minerals, collapsed at temperatures below 600 °C. This behavior is consistent with findings reported in the literature, where kaolinite typically undergoes dehydroxylation and transforms into an amorphous metakaolin phase at approximately 500–600 °C, as previously observed by Castelein et al. (2001) [32] and Ponce and Irassar (2008) [33]. Chlorite, on the other hand, also experiences structural destabilization within a similar temperature range due to the loss of hydroxyl groups, as reported by Hajjaji et al. (2010) [34].

In addition to clay mineral decomposition, the XRD analysis indicated the breakdown of carbonates, such as calcite and dolomite, at temperatures below 900 °C. These observations are consistent with earlier studies by Resio (2024) [35] and M Milošević (2024) [36], where calcite decomposition typically occurs between 700 and 900 °C, producing lime (CaO) and carbon dioxide. Dolomite, being a double carbonate of calcium and magnesium, decomposes in two stages, beginning at approximately 700 °C and completing at approximately 900 °C, yielding both CaO and MgO. The decomposition of these carbonates plays a critical role in releasing lime (CaO), which actively participates in subsequent phase formation.

**Figure 4:**
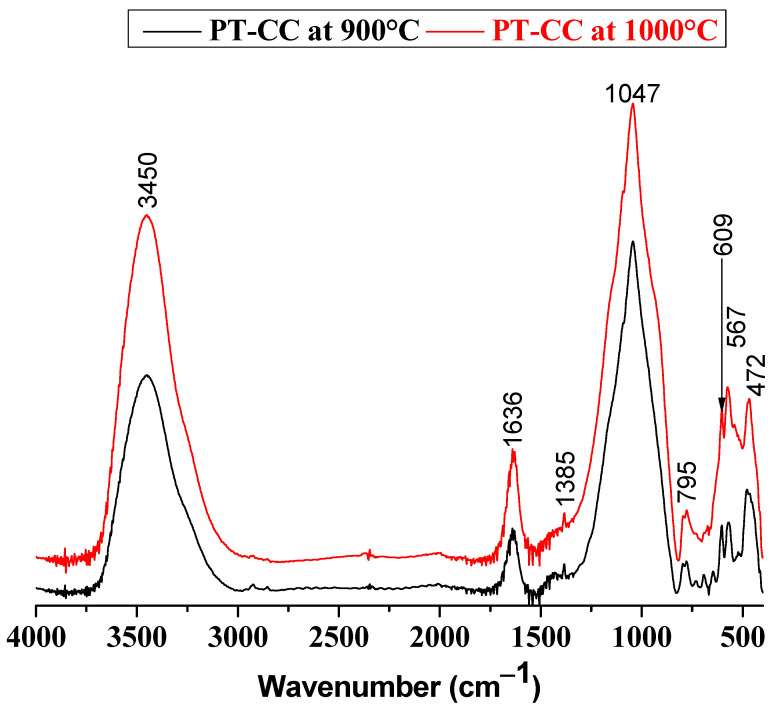
FTIR spectra of membranes of the studied blends heated at 900 °C and 1000 °C.

At 900 °C and beyond (Figure 3b), the emergence of labradorite, a calcium-sodium aluminosilicate feldspar, was observed, which is attributed to the reaction between the lime released from carbonate decomposition and the amorphous aluminosilicate phases derived from the breakdown of kaolinite and chlorite. This result closely aligns with the phase equilibria predictions shown in Figure 4 and parallels findings from studies on clay-carbonate mixtures, where feldspar phases such as labradorite and anorthite are often formed at temperatures above 850 °C [37,38]. Labradorite formation underscores the interaction between decomposed carbonates and silica-alumina sources, which is a critical step in developing new crystalline phases within ceramic systems.

Interestingly, despite theoretical predictions suggesting the presence of wollastonite (CaSiO_3_) under these conditions, this phase was notably absent. The absence of wollastonite can likely be attributed to a lack of sufficient contact or reactivity between quartz and lime within the system. Previous studies, such as those by Elimbi et al. (2011) [39], have shown that wollastonite formation requires intimate mixing and optimal temperature conditions to facilitate the necessary solid-state reaction between lime and silica. The heterogeneous distribution of components in the PT-CC blend may have limited this interaction, resulting in the preferential formation of labradorite instead.

The mineralogy of the membranes has not been altered by the deposition of the geopolymetric thin layer, as the amount of material added is minimal in terms of quantity. This is why the results for these materials were not provided, as they yield the same outcomes as those without any deposition.

**Figure 5:**
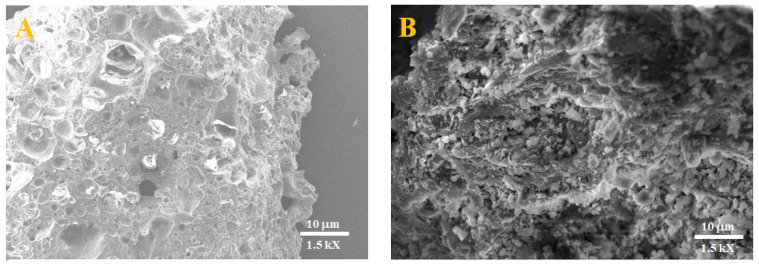
SEM micrographs of the manufactured membranes at 1000 °C. (**A**): PT-CC free of spraying and (**B**): PT-CC with spraying.

The SEM micrographs (Figure 5) illustrate the surface morphology of ceramic membranes fabricated at 1000 °C, comparing a sample without spraying (A) to one with spraying (B). The membrane without spraying (A) exhibits a highly porous structure with visible voids and interconnected pores, resulting from the natural characteristics of the precursor material and the sintering process. However, the larger and less uniform pores may limit the membrane’s performance in terms of selectivity and mechanical strength. In contrast, the sprayed membrane (B) displays a denser and more compact structure, with reduced and more uniform pore sizes. The spraying process appears to have introduced finer particles or coatings that fill the larger voids, effectively tailoring the porosity. This modification enhances the membrane’s functional properties, such as filtration efficiency and mechanical robustness, making it more suitable for advanced applications. Further testing, such as permeability or durability analysis, would confirm these enhancements.

### 3.3. Effect of Geopolymer Coating on Membrane Performance

**Figure 6:**
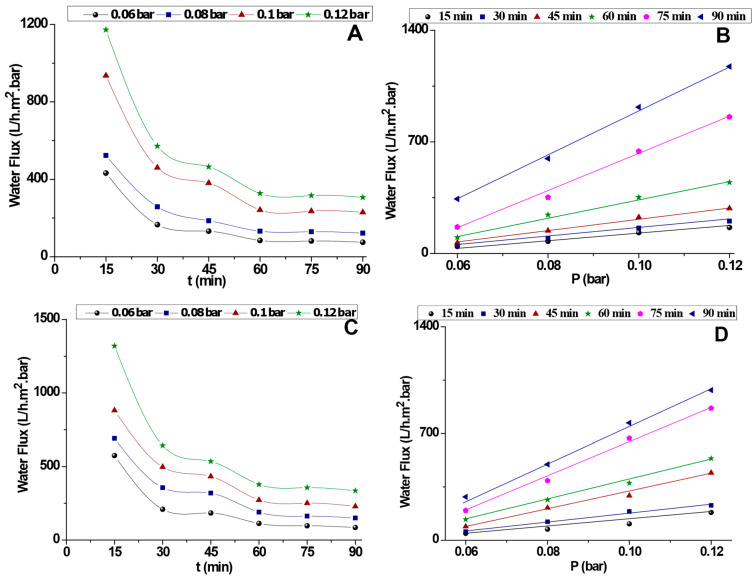
Filtration performances through variation of the permeation flux of an industrial effluent as a function of time and applied pressure. (**A**,**B**): PT-CC; (**C**,**D**): PT-CC sprayed with alkali-activated materials.

Figure 6 presents the filtration performances of an industrial effluent by examining the permeation flux over time and as a function of applied pressure for the two prepared membranes.

In Figure 6A, the variation of water flux with time for PT-CC at different applied pressures demonstrates a rapid decline in flux during the first 30 min. This behavior can be attributed to membrane fouling caused by the accumulation of particles from the industrial effluent, which gradually blocks the membrane pores and reduces its permeability. Although higher applied pressures, such as those shown in curves (d) and (c), initially result in higher water flux, the rate of decline stabilizes over time and tends to approach similar values observed at lower applied pressures. This suggests that fouling dominates the filtration process as time progresses, regardless of the applied pressure.

In Figure 6A, water flux is plotted against the applied pressure for PT-CC. The flux exhibits a linear increase with increasing pressure, indicating that the membrane operates without severe pressure-induced limitations within the tested range (0.06–0.12 bar). Among the curves, the higher performance levels, labeled (f) and (e), exhibit the greatest water flux values, highlighting that under specific conditions, the PT-CC membrane can achieve improved permeation efficiency. This linearity reflects the absence of critical fouling or membrane compaction within the selected operational pressures.

For PT-CC membranes modified with alkali-activated materials (Figure 6C,D), significant improvements in water flux are observed. In Figure 6A, water flux as a function of time demonstrates considerably higher initial values across all applied pressures when compared to the unmodified PT-CC membranes (Figure 6A). This improvement can be attributed to enhanced hydrophilicity, better pore structure, or reduced resistance to water permeation as a result of the alkali-activated coating. However, as with the unmodified PT-CC membranes, water flux decreases sharply during the first 30 min, likely due to fouling caused by the industrial effluent. Nonetheless, the stabilized flux values remain higher than those for the unmodified membranes, indicating the improved performance of the coated PT-CC. Notably, at higher pressures, such as curve (d), the water flux remains superior, reinforcing the positive impact of the alkali-activated material.

In Figure 6D, the relationship between water flux and applied pressure for the modified PT-CC membranes again reveals a linear increase, similar to the trend observed in Figure 6B. However, the overall water flux values are consistently higher for the alkali-activated membranes compared to the unmodified PT-CC. Curves (e) and (f) exhibit the highest flux values, demonstrating that the alkali-activated material enhances the membrane’s capacity for water permeation under varying pressures. This improved performance can be attributed to the modification’s ability to optimize pore structure and reduce fouling resistance, enabling better flux under applied pressure.

In summary, the comparison between PT-CC and PT-CC sprayed with alkali-activated materials highlights the significant enhancement in filtration performance achieved through membrane modification. The alkali-activated coating effectively increases water flux and improves resistance to fouling, as evidenced by the higher permeation values over time and under varying pressures. These findings underscore the potential of alkali-activated materials as a promising strategy for enhancing membrane performance in the treatment of industrial effluents.

Table 2 compares the characteristics of textile effluent before and after filtration, revealing significant changes in key parameters. The pH of the effluent decreases slightly after filtration, from 7.8 before filtration to 7.23 after filtration by PT-CC and 6.99 after filtration by sprayed PT-CC, indicating a mild acidification following treatment. Conductivity, which reflects the concentration of dissolved ions, also drops from 690 µS/cm before filtration to 671 µS/cm after PT-CC filtration and further to 659 µS/cm after sprayed PT-CC filtration, suggesting a reduction in ion concentration. Turbidity, a measure of suspended particles, decreases dramatically, from 152 NTU before filtration to 1.09 NTU after PT-CC filtration and 0.89 NTU after spraying PT-CC filtration, showing the efficiency of both filtration methods in removing suspended solids. Absorbance at 664 nm, which indicates the presence of organic compounds, also declines from 0.121 before filtration to 0.093 and 0.079 after PT-CC and sprayed PT-CC filtration, respectively, pointing to a reduction in colored substances. The removal efficiency (RT%) shows a marked improvement, increasing from 0% before filtration to 95.6% after PT-CC and 98.9% after spraying PT-CC filtration, highlighting the effectiveness of both methods in pollutant removal. Finally, the relative absorbance (RABS%) increases from 0% to 27% and 31% after PT-CC and sprayed PT-CC filtration, respectively, indicating enhanced removal of organic compounds and color. Overall, the filtration processes, particularly the sprayed PT-CC method, significantly improve the quality of the textile effluent by reducing turbidity, conductivity, absorbance, and increasing removal efficiency.

**Figure 7:**
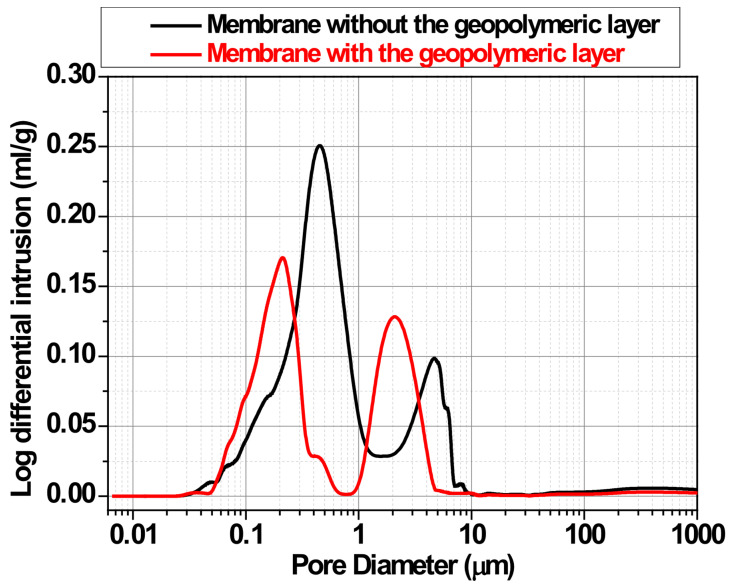
Pore size of the synthesized membranes prior and after spraying the activated materials.

Figure 7 displayed the pore size of the synthesized membranes prior and after spraying the activated materials. The pore size distribution of the clay-based ceramic membranes, both before and after the application of a geopolymeric layer, highlights the significant impact of the coating process on the membrane’s microstructure. As seen in the figure, the uncoated membrane (black curve) exhibits a dominant pore size range of approximately 1–10 µm, characteristic of clay-based ceramics. This broad distribution reflects the natural porosity of the sintered ceramic matrix, which arises from the volatilization of organic phases and the sintering of clay particles during thermal treatment.

In contrast, the application of a geopolymeric layer (red curve) results in a marked shift toward smaller pore sizes, predominantly in the range of 0.1–1 µm. This shift can be attributed to the penetration of the geopolymeric slurry into the ceramic matrix, effectively filling larger pores and forming a denser surface layer. The geopolymeric material, rich in aluminosilicate gel phases, polymerizes and adheres tightly to the ceramic surface, thereby reducing the effective pore size. This densification process not only alters the pore structure but also enhances the membrane’s mechanical and chemical stability, making it suitable for high-performance filtration applications.

Moreover, the geopolymeric coating leads to a reduction in total pore volume, evidenced by the decreased intensity of the intrusion curve. The narrower pore size distribution of the coated membrane indicates improved uniformity, which is critical for filtration applications, as it minimizes the risk of fouling and ensures consistent particle retention. Such structural modifications are essential for tailoring membranes to meet specific industrial requirements, such as ultrafiltration or microfiltration.

From a functional perspective, the reduced pore size and volume after coating with a geopolymeric layer enhance the membrane’s filtration capacity, particularly for fine particles and contaminants in water treatment. Several studies have demonstrated that membranes with smaller and more uniform pore sizes exhibit superior performance in rejecting suspended solids, organic pollutants, and bacteria. The figure supports these findings, indicating that the geopolymer-coated membrane would likely outperform its uncoated counterpart in such applications.

**Figure 8:**
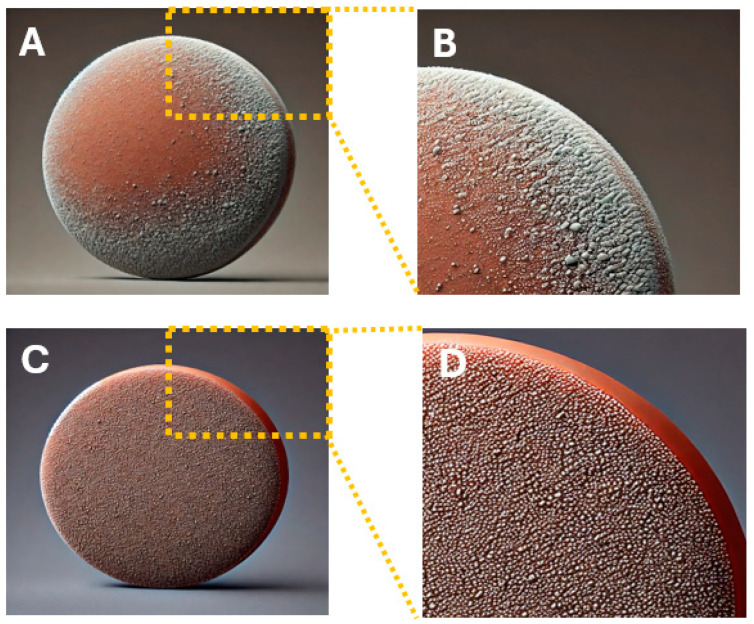
Views of synthesized membranes prior and after spraying the activated materials. (**A**,**B**): Membranes prior spraying. (**C**,**D**): mambranes after spraying. (**B**,**D**): magnifications of A and C respectively.

Figure 8 presents optical images of membrane surfaces before and after deposition, providing a comparative analysis of two types of membrane supports. The images highlight the morphological differences observed due to the deposition process.

After deposition, significant changes were observed within the manufactured membrane. The membrane surface exhibits scattered particles and noticeable variations in color and texture, indicating a rougher and less uniform appearance compared to its original state. The color of the membrane after modification becomes mottled, with clear variations in tone, suggesting an uneven deposition of the particles. These observations emphasize the impact of the deposition process on the membrane surfaces.

Table 3 summarizes the compressive strength of elaborated ceramic membranes, both with and without a geopolymeric separation layer. The results indicate that the membranes with the geopolymeric separation layer exhibit a higher average compressive strength compared to those without. This suggests that the geopolymeric layer enhances the overall mechanical stability of the membranes. The lower standard deviation for the membranes with the separation layer also indicates a greater consistency in their compressive strength. The increased compressive strength of the membranes with the geopolymeric layer could be attributed to several factors: (i) Improved interfacial bonding: The geopolymeric layer may create a stronger bond between the membrane and the support structure, leading to better stress distribution and enhanced load-bearing capacity. (ii) Reduced porosity: The geopolymeric layer might fill in pores or defects in the membrane structure, effectively reducing porosity and increasing the density and strength of the material.

**Figure 9:**
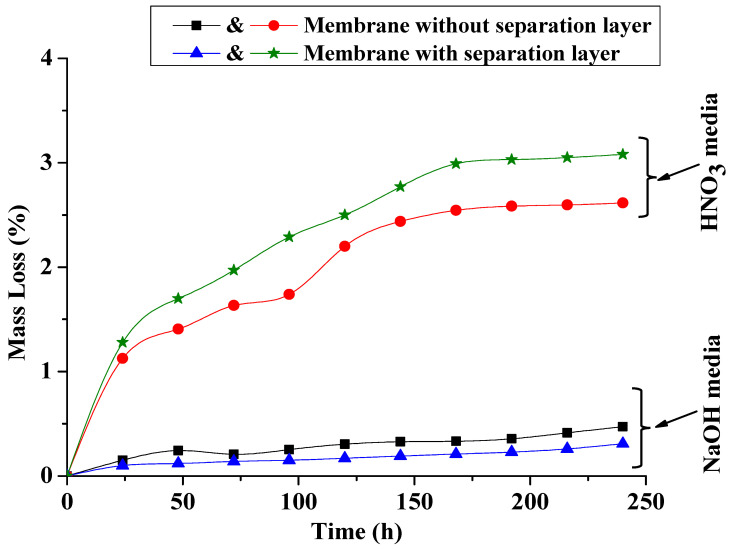
Weight loss of the produced membranes in nitric acid (pH = 1) and sodium hydroxide (pH = 13) solution against time (h) at ambient temperature.

Figure 9 depicts the mass loss of clay-based ceramic membranes, both with and without a geopolymeric separation layer, when exposed to acidic (HNO_3_, pH = 1) and alkaline (NaOH, pH = 13) solutions over time. Notably, the membranes without the separation layer exhibit a significant mass loss in the acidic solution, indicating degradation and dissolution of the membrane material. Conversely, the membranes with the geopolymeric layer demonstrate considerably lower mass loss in the acidic environment, suggesting that the geopolymeric layer acts as a protective barrier, shielding the underlying membrane material from the aggressive acidic solution. In the alkaline solution, both types of membranes show negligible mass loss, highlighting the overall robustness of the membranes in this environment. These findings strongly suggest that the geopolymeric separation layer significantly enhances the chemical durability of the clay-based ceramic membranes, particularly in acidic conditions, making them more suitable for applications where they are exposed to harsh chemical environments.

## 4. Conclusions

This study demonstrates the feasibility and effectiveness of utilizing phosphate tailings and clay lithologies as sustainable raw materials for ceramic membrane production. By integrating alkali-activated coatings, the porosity and structural properties of the membranes were successfully tailored, resulting in enhanced filtration performance without compromising mechanical or physicochemical stability. The membranes exhibited excellent performance in wastewater filtration tests, validating their suitability for environmental and industrial applications.

The results underline the potential of combining waste-derived materials with advanced geopolymerization techniques to develop cost-effective and environmentally friendly ceramic membranes. This approach not only addresses the environmental challenges posed by the disposal of phosphate tailings and clay residues but also aligns with circular economy principles by transforming industrial byproducts into high-value filtration materials.

Future research could focus on scaling up this process, optimizing coating uniformity, and exploring the long-term operational performance of these membranes in various industrial settings. By advancing the development of sustainable ceramic membranes, this study contributes to addressing the global need for efficient water treatment technologies, supporting both environmental preservation and resource efficiency.

## Figures and Tables

**Table 1:** Mineralogical and chemical compositions (% mass) of both phosphate tailings and chloritic clay.

Chemical Composition										
Sample	SiO_2_	Al_2_O_3_		Fe_2_O_3_	MgO	CaO		Na_2_O	K_2_O	P_2_O_5_
PT	28.5	3.1		1.1	5.1	42.7		0.9	0.5	17.5
CC	53.2	18.5		6.4	2.2	1.7		1.8	4.4	-
Mineralogical Composition										
Sample	I	K	Ch	M	F	Q	D	C	H	
PT	-	-	-	-	44	17	7	15	-	
CC	33	25	10	-	-	19	6	-	7	

F: fluorapatite; Q: quartz; D: dolomite; C: calcite; H: hématite.

**Table 2:** Comparison of textile effluent characteristics prior to and following filtration.

Sample	State	PH	Conductivity (mS/cm)	Turbidity	Absorbance at 664 nm	R_T_ (%)	R_ABS_ (%)
Textile effluent	Before filtration	7.8	690	152	0.121	0	0
	After filtration by PT-CC	7.23	671	1.09	0.093	95.6	27
	After filtration by sprayed PT-CC	6.99	659	0.89	0.079	98.9	31

**Table 3:** Compressive strength of the produced membranes prior to and after spraying the separation layer.

	Membrane Without Separation Layer		Membrane with Separation Layer	
Compressive strength (MPa)	Value	Standard deviation	Value	Standard deviation
	19.373	1.067	20.045	0.876

## Data Availability

Data will be provided on request.

## Abbreviations

The following abbreviations are used in this manuscript:

CC	Chloritic clay
CB	Cork oak bark
COD	Chemical oxygen demand
FTIR	Fourier transform infra-red
PT	Phosphate tailings
SEM	Scan electron microscope
TGA	Thermogravimetric analysis
NaOH	Sodium hydroxide
Na_2_SiO_3_	Sodium silicate
XRF	X-ray fluorescence
XRD	X-ray diffraction
